# Comparative efficacy, safety, and functional recovery of neonatal fc receptor inhibitors and conventional immunotherapies for chronic inflammatory demyelinating polyradiculoneuropathy: a systematic review and network meta-analysis

**DOI:** 10.3389/fimmu.2026.1856507

**Published:** 2026-09-14

**Authors:** Qingqing Jiang, Zhengtian Gu

**Affiliations:** First People’s Hospital of Changzhou, Changzhou, China

**Keywords:** chronic inflammatory demyelinating polyradiculoneuropathy, CIDP, corticosteroids, efgartigimod alfa, FcRn inhibitors, intravenous immunoglobulin, network meta-analysis, plasma exchange

## Abstract

**Background:**

Chronic inflammatory demyelinating polyradiculoneuropathy (CIDP) management is undergoing a paradigm shift with the emergence of targeted biologicals. We aimed to compare the efficacy, safety, and functional outcomes of neonatal Fc receptor (FcRn) inhibitors against conventional immunotherapies.

**Methods:**

We conducted a systematic review and frequentist network meta-analysis (NMA) of randomized controlled trials (RCTs) indexed in PubMed/MEDLINE, Embase, CENTRAL, Web of Science, ClinicalTrials.gov, and WHO ICTRP from database inception to February 2026. Interventions included FcRn inhibitors (efgartigimod alfa, rozanolixizumab), intravenous/subcutaneous immunoglobulin (IVIg/SCIG), corticosteroids, plasma exchange (PLEX), and rituximab. The primary outcome was clinical response rate. Secondary outcomes included serious adverse events (SAEs), discontinuation due to adverse events (DCAE), and grip-strength change as a distal motor outcome. Treatments were ranked using P-scores, and certainty of evidence was summarized using CINeMA/GRADE domains.

**Findings:**

Eighteen RCTs (n=2,184) were included. In the primary efficacy network (I2 = 0.37%), PLEX (OR 33.60, 95% CI 3.15–358.90; P-score 0.9415) and corticosteroids (OR 15.44, 95% CI 3.53–67.47; P-score 0.9056) demonstrated the highest probability of achieving clinical response. Efgartigimod alfa showed significant clinical response (OR 3.14, 95% CI 1.34–7.33) and ranked highest for grip-strength change (SMD 0.67, 95% CI 0.09–1.24; P-score 0.8847), although this analysis was based on five studies and should be interpreted as a distal motor outcome rather than a comprehensive functional endpoint. For secondary outcomes, FcRn inhibitors had SAE estimates comparable to placebo (efgartigimod OR 1.00, 95% CI 0.31–3.20; rozanolixizumab OR 0.94, 95% CI 0.05–16.37), and rozanolixizumab ranked highest for DCAE/acceptability (P-score 0.7896). Heterogeneity was low to modest across secondary networks (SAEs I2 = 12.40%; DCAE I2 = 0.00%; grip strength I2 = 24.10%).

**Interpretation:**

PLEX and corticosteroids showed the strongest relative effects for short-term clinical response, whereas efgartigimod alfa showed a significant treatment effect and the highest ranking for grip-strength change among studies reporting this distal motor measure. IVIg remains a broadly supported comparator across induction and maintenance settings. These findings should inform, but not replace, individualized clinical decision-making because patient-level treatment-effect modification, biomarker status, prior treatment response, and long-term neurophysiological outcomes were not directly evaluated.

## 1 Introduction

Chronic inflammatory demyelinating polyradiculoneuropathy (CIDP) is a rare, heterogeneous, and debilitating immune-mediated disorder of the peripheral nervous system (1). It is characterized by progressive or relapsing symmetrical proximal and distal muscle weakness, sensory disturbances, and diminished or absent deep tendon reflexes, typically evolving over a period of at least eight weeks (2). Pathophysiologically, CIDP involves a complex interplay between cellular and humoral immunity, resulting in inflammatory demyelination of nerve roots and peripheral nerves (3). The latest clinical guidelines from the European Academy of Neurology and Peripheral Nerve Society (EAN/PNS) emphasize the importance of accurate diagnosis to distinguish typical CIDP from its variants and autoimmune nodopathies associated with specific IgG4 antibodies (4).

For several decades, the therapeutic landscape for CIDP has been dominated by three established first-line treatments: intravenous immunoglobulin (IVIg), corticosteroids, and plasma exchange (PLEX) (5). Randomized controlled trials have consistently demonstrated the efficacy of these interventions in achieving short-term clinical remission and disability reduction (6). However, the choice of initial therapy remains largely empirical, as head-to-head evidence comparing their long-term superiority is limited (7). While approximately 70% to 80% of patients respond to these traditional immunotherapies, a significant proportion requires chronic, repeated dosing to maintain functional stability (8).

Despite their efficacy, conventional therapies carry substantial limitations that impact patient quality of life and long-term prognosis. Long-term corticosteroid use is frequently associated with systemic adverse effects, such as osteoporosis, metabolic disturbances, and psychiatric complications (9). IVIg, while generally better tolerated, imposes a high treatment burden due to its cost, the requirement for frequent intravenous infusions, and potential complications like venous thromboembolism (10). PLEX, though highly effective in refractory cases, is technically demanding and restricted by the need for specialized equipment and venous access (11). Furthermore, a subset of patients remains refractory to all three first-line agents, necessitating the exploration of second-line immunosuppressants like rituximab, although robust clinical trial data for these agents remain sparse (12).

The recent elucidation of the role of pathogenic IgG antibodies in CIDP has catalyzed the development of more targeted biological interventions (13). A pivotal advancement is the emergence of neonatal Fc receptor (FcRn) inhibitors. By blocking the FcRn-IgG interaction, these agents accelerate the clearance of circulating IgG, including pathogenic autoantibodies, without affecting the levels of other immunoglobulins or the innate immune response (14). The pivotal Phase 3 ADHERE trial for efgartigimod alfa demonstrated significant efficacy in reducing the risk of clinical relapse, marking a potential paradigm shift in the management of CIDP (15). Similarly, rozanolixizumab has shown promise in early-phase studies, suggesting that FcRn antagonism could become a cornerstone of future therapeutic strategies (16).

Furthermore, the transition from intravenous to subcutaneous administration of immunoglobulin (SCIG) has offered a more convenient maintenance option, providing stable plasma levels and reducing systemic side effects (17). The introduction of B-cell depletion therapies and next-generation immunomodulators further complicates the decision-making process for clinicians (18). In the absence of direct head-to-head trials comparing these novel biologics with established first-line therapies, determining the optimal therapeutic sequence and the comparative efficacy and safety profile of these agents remains a critical unmet need in neuromuscular medicine (19).

Network meta-analysis (NMA) offers a powerful statistical framework to integrate direct and indirect evidence from multiple randomized controlled trials, enabling a comprehensive comparison of all available interventions (20). Given the rapid influx of clinical data through 2024 and 2025, a systematic synthesis is essential to guide evidence-based practice. This study aims to conduct a systematic review and NMA to evaluate the comparative efficacy, safety, and tolerability of FcRn inhibitors, SCIG, IVIg, corticosteroids, and PLEX in adult patients with CIDP. By incorporating the latest functional outcomes, such as grip strength and clinical response rates, we seek to identify the intervention that provide the optimal balance between clinical benefit and user-acceptability.

## 2 Methods

### 2.1 Search strategy and selection criteria

We conducted a systematic search of several electronic databases, including PubMed/MEDLINE, Embase, the Cochrane Central Register of Controlled Trials (CENTRAL), and Web of Science, from their inception through February 2026. The search employed a combination of medical subject headings (MeSH) and free-text terms related to “chronic inflammatory demyelinating polyradiculoneuropathy”, “immunoglobulin”, “corticosteroids”, “plasma exchange”, and “neonatal Fc receptor inhibitors”. To ensure a comprehensive evidence base, we also screened clinical trial registries (ClinicalTrials.gov and the WHO International Clinical Trials Registry Platform) and the reference lists of previous systematic reviews.

Studies were eligible for inclusion if they met the following criteria: (1) randomized controlled trials (RCTs) involving adult patients (aged ≥ 18 years) diagnosed with “definite” or “probable” CIDP according to established criteria (e.g., EFNS/PNS 2010 or 2021, AAN); (2) evaluation of at least one of the following interventions: FcRn inhibitors (e.g., efgartigimod, rozanolixizumab), IVIg, SCIG, corticosteroids, PLEX, or rituximab; and (3) report of at least one clinical efficacy, safety, or functional outcome. We excluded non-randomized studies, pilot studies with insufficient data, and trials focusing on other polyneuropathies (e.g., multifocal motor neuropathy).

### 2.2 Data extraction and quality assessment

Two reviewers independently performed study selection, and a third reviewer resolved any discrepancies. We extracted structured data using a standardized form, including study characteristics (author, year, trial phase, design), patient demographics (age, sex, disease duration), intervention details (dose, frequency, route of administration), and outcome metrics.

The methodological quality of the included RCTs was assessed using the Cochrane Risk of Bias 2.0 (RoB 2.0) tool. We evaluated five specific domains: bias arising from the randomization process, bias due to deviations from intended interventions, bias due to missing outcome data, bias in measurement of the outcome, and bias in selection of the reported result. Each domain and the overall risk of bias were categorized as “low risk”, “some concerns”, or “high risk”.

### 2.3 Outcome measures

The primary efficacy outcome was the Clinical Response Rate, defined as the proportion of patients achieving a significant improvement in disability (e.g., a decrease of ≥ 1 point on the Inflammatory Neuropathy Cause and Treatment [INCAT] scale or the Overall Neuropathy Limitation Scale [ONLS]) or the prevention of relapse during a randomized withdrawal phase.

Secondary outcomes included: (1) safety, measured by the incidence of serious adverse events (SAEs); (2) treatment acceptability, defined as discontinuation due to adverse events (DCAE); and (3) grip-strength change, treated as a distal motor impairment measure rather than a stand-alone surrogate for multidimensional functional recovery. Disability scales (INCAT, ONLS, I-RODS), relapse prevention, gait function, and patient-reported outcomes were considered when reported but were too heterogeneously available for a unified functional NMA.

### 2.4 Statistical analysis

We performed a frequentist random-effects network meta-analysis (NMA) to integrate direct and indirect evidence. For binary outcomes (Clinical Response, SAEs, DCAE), we calculated Odds Ratios (ORs) with 95% confidence intervals (CIs). For the continuous outcome (Grip Strength), we calculated the Standardized Mean Difference (SMD) to account for different measurement tools across studies.

The transitivity assumption was assessed by comparing potential effect modifiers across direct comparisons, including trial era, design (parallel versus crossover), treatment phase, prior treatment exposure, disease duration, baseline disability, sample size, and risk of bias (**Supplementary Table 5**). We ranked the interventions using P-scores, which range from 0 to 1, with higher values indicating a higher probability of the treatment being most effective or most favorable for the outcome. P-scores from sparse networks were interpreted descriptively rather than as definitive clinical rankings.

Statistical heterogeneity within each network was evaluated using the I2 statistic and is reported for clinical response, SAEs, DCAE, and grip strength. Global inconsistency was assessed using the design-by-treatment interaction model (Cochran’s Q), while local inconsistency was evaluated using the node-splitting method where data allowed. Clinical phase was classified operationally before analysis: induction trials enrolled treatment-naive or actively deteriorating patients receiving active short-term therapy; maintenance/withdrawal trials enrolled treatment-responsive, stable, or immunoglobulin-dependent patients randomized to continue, withdraw, or switch maintenance therapy. Mixed trials were classified according to the randomized phase contributing outcome data and were examined in sensitivity analyses. Small-study effects and potential publication bias were assessed using comparison-adjusted funnel plots and Egger’s regression test. All analyses were conducted in R using netmeta for the primary NMA; revised participant-scaled network and grip-strength contribution plots were regenerated in R from the extracted arm-level dataset.

## 3 Results

### 3.1 Study selection and characteristics

The initial database search yielded 858 records. After removing duplicates and screening titles and abstracts, 70 reports were assessed for full-text eligibility. Ultimately, 18 randomized controlled trials (RCTs) involving adult patients with CIDP were included in this systematic review and network meta-analysis (**Figure 1**).

**Figure 1:**
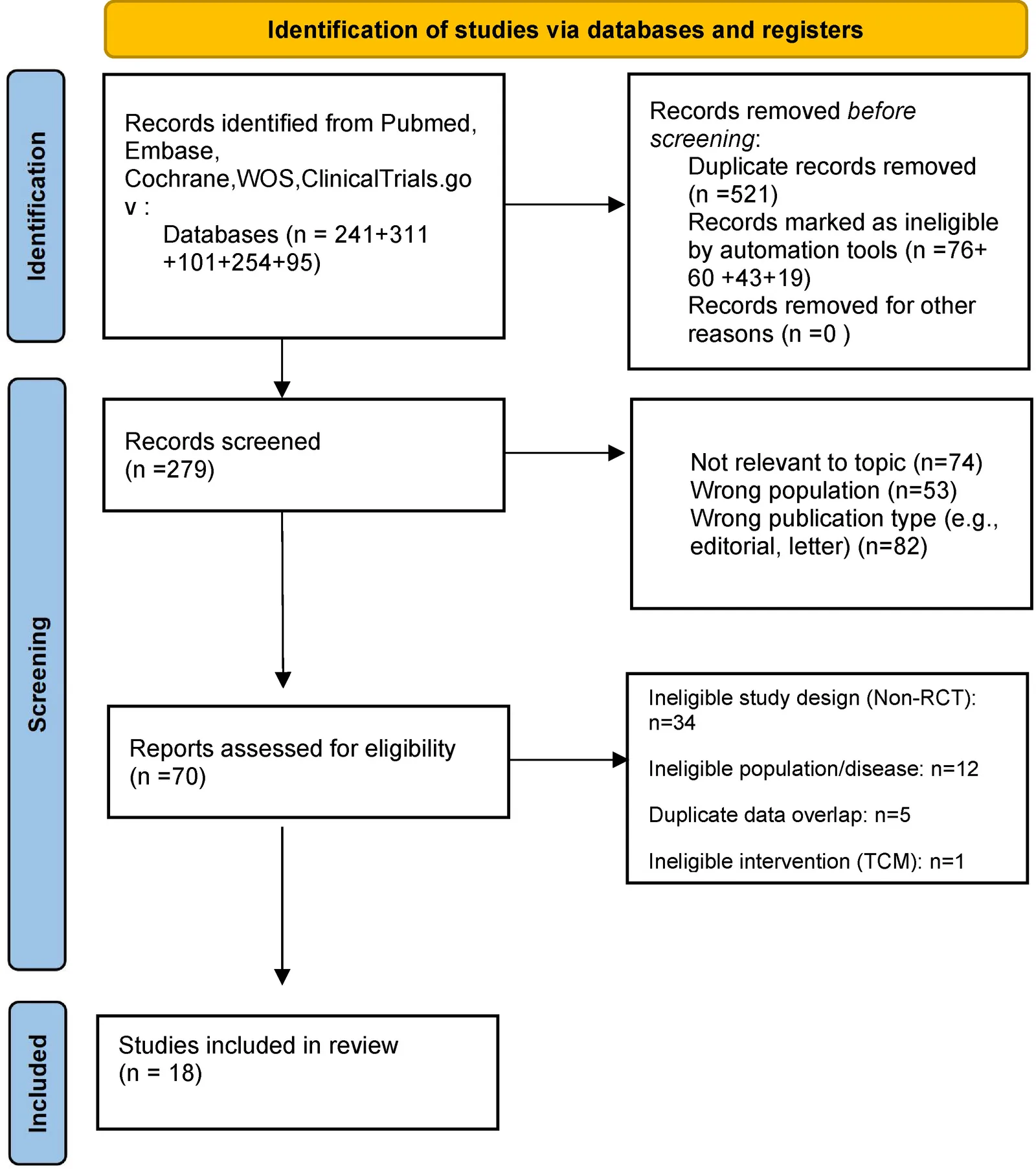
PRISMA 2020 flow diagram of the study selection process. The flow diagram illustrates the systematic literature search and screening process.

The 18 included studies (21–38) were published between 1994 and 2026. These trials evaluated a wide spectrum of immunotherapies, including plasma exchange (PLEX) (21), intravenous immunoglobulin (IVIg) (22–25, 27, 28, 31, 33–35), subcutaneous immunoglobulin (SCIG) (29–32), corticosteroids (24, 26, 28), and novel biologics such as the FcRn inhibitors efgartigimod alfa (37) and rozanolixizumab (36), and the B-cell depleting agent rituximab (38). The trials covered both treatment-naïve patients (Induction phase) and those on stable maintenance therapy (Maintenance/Withdrawal phase). Detailed characteristics are summarized in **Table 1**.

**Table 1:** Characteristics of the 18 randomized controlled trials included in the network meta-analysis.

Study ID (author, year)	Population	Intervention(s)	Comparator	Total N	Duration (wks)	Primary outcome
Dyck, 1994 (21)	Naïve	PLEX	Sham	32	6	MRC Score
Hahn, 1996 (22)	Mixed	IVIg (Gamimune)	Placebo	30	4	INCAT
Mendell, 2001 (23)	Naïve	IVIg (Gamunex)	Placebo	53	6	Muscle Strength
Hughes, 2001 (24)	Naïve	IVIg vs. Prednisolone	Head-to-head	60	12	Disability Score
Merkies, 2009 (ICE) (25)	Mixed	IVIg (Gamunex)	Placebo	117	24	INCAT
van Schaik, 2010 (PREDICT) (26)	Naïve	Pulsed Dexamethasone	Daily Prednisolone	40	52	ONLS
Kuitwaard, 2010 (27)	Maintenance	IVIg (Gammagard)	Kiovig	27	10	ODSS
Nobile-Orazio, 2012 (28)	Naïve	IVIg	IV Methylpred	45	24	INCAT
Markvardsen, 2013 (29)	Maintenance	SCIG	Placebo	30	12	Muscle Strength
Van Schaik, 2017 (PATH) (30)	Maintenance	SCIG (Low/High)	Placebo	172	24	Relapse Rate
Markvardsen, 2017 (31)	Naïve	SCIG	IVIg	20	20	Muscle Strength
Hughes, 2018 (32)	Maintenance	SCIG (Novartis)	Placebo	106	24	INCAT
Kuitwaard, 2021 (33)	Maintenance	High-freq IVIg	Standard IVIg	25	24	Muscle Strength
Cornblath, 2022 (FORZA) (34)	Maintenance	IVIg (Low/High)	IVIg (Mid-dose)	142	24	Relapse Rate
Adrichem, 2022 (35)	Maintenance	IVIg Continuation	IVIg Withdrawal	60	24	INCAT
Querol, 2024 (36)	Maintenance	Rozanolixizumab	Placebo	34	13	Relapse Rate
Allen, 2024 (ADHERE) (37)	Maintenance/withdrawal (Stage A responders; mixed prior treatment exposure)	Efgartigimod alfa	Placebo	221	48	Relapse Rate
Nobile-Orazio, 2025 (38)	Naïve	Rituximab	Placebo	37	52	INCAT

Summary of the 18 primary clinical trials included in the systematic review.

### 3.2 Risk of bias assessment

Overall, most included trials were judged at low risk of bias by RoB 2.0, but this finding does not remove uncertainty introduced by older crossover designs, small pilot trials, and randomized-withdrawal designs. Earlier crossover trials may be vulnerable to carry-over effects and period effects despite washout procedures, small pilot trials contribute imprecise estimates, and withdrawal designs enrich the randomized population for prior responders, which may limit generalizability to treatment-naive patients. These issues were incorporated into the certainty-of-evidence assessment and are now explicitly considered when interpreting sparse nodes such as rozanolixizumab and rituximab (**Supplementary Table 6**).

### 3.3 Primary outcome: clinical response

The network meta-analysis (NMA) for clinical response included 11 direct comparisons from the extracted responder dataset. As shown in the revised network plot (**Figure 2A**), node size is proportional to the number of randomized participants contributing responder data and edge thickness reflects the number of direct comparisons. PLEX, corticosteroids, and IVIg served as the central conventional nodes, whereas rozanolixizumab and rituximab remained sparsely connected.

**Figure 2:**
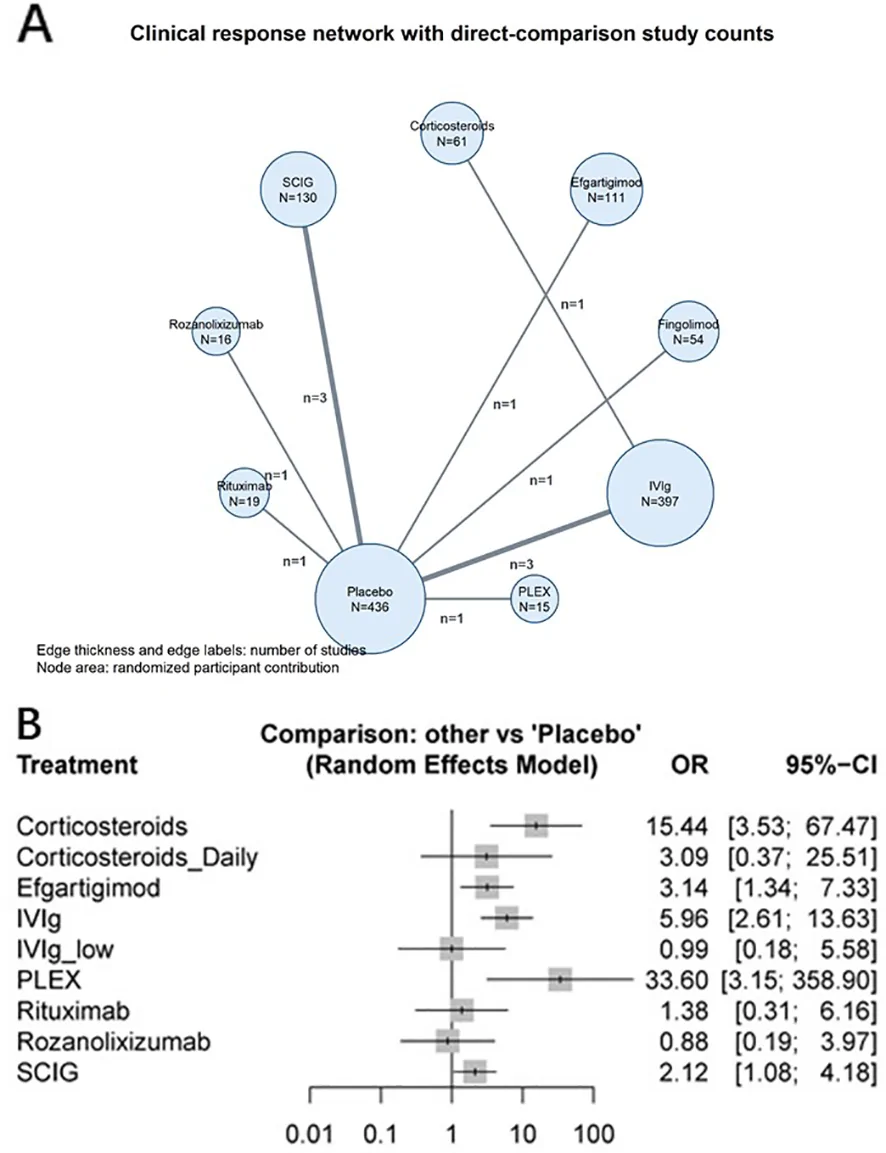
Primary efficacy analysis (clinical response). **(A)** Revised network plot of treatment comparisons for clinical response. Node size is proportional to the number of randomized participants contributing responder data, and edge thickness represents the number of direct comparisons for each head-to-head contrast. **(B)** Forest plot of clinical response. Results are expressed as Odds Ratios (OR) with 95% Confidence Intervals (CI) compared to Placebo. Treatments are sorted by P-scores. PLEX and Corticosteroids demonstrate the highest relative efficacy in terms of disability improvement.

Compared to placebo, all active interventions except rozanolixizumab and rituximab showed significant disability improvement. PLEX (21) demonstrated the highest relative efficacy (OR 33.60; 95% CI 3.15–358.90), followed by corticosteroids (OR 15.44; 95% CI 3.53–67.47) and IVIg (OR 5.96; 95% CI 2.61–13.63). Among novel therapies, efgartigimod alfa (37) showed a significant clinical response (OR 3.14; 95% CI 1.34–7.33), and SCIG (17, 32) also remained effective (OR 2.12; 95% CI 1.08–4.18) (**Figure 2B**). According to P-scores, the treatment ranking for efficacy was PLEX (0.9415) > Corticosteroids (0.9056) > IVIg (0.7392) > Efgartigimod (0.5668) > SCIG (0.4427) (Appendix Table 1).

### 3.4 Secondary outcomes: safety and acceptability

Safety was evaluated by the incidence of serious adverse events (SAEs) (**Figure 3A**). The SAE network showed low heterogeneity (I2 = 12.40%) and no significant global inconsistency (Q-test P = 0.642). Daily corticosteroids showed the lowest point estimate for SAE risk (OR 0.12; 95% CI 0.00–11.52), while FcRn inhibitors demonstrated estimates comparable to placebo, with efgartigimod at an OR of 1.00 (95% CI 0.31–3.20) and rozanolixizumab at an OR of 0.94 (95% CI 0.05–16.37) (**Figure 3B**).

**Figure 3:**
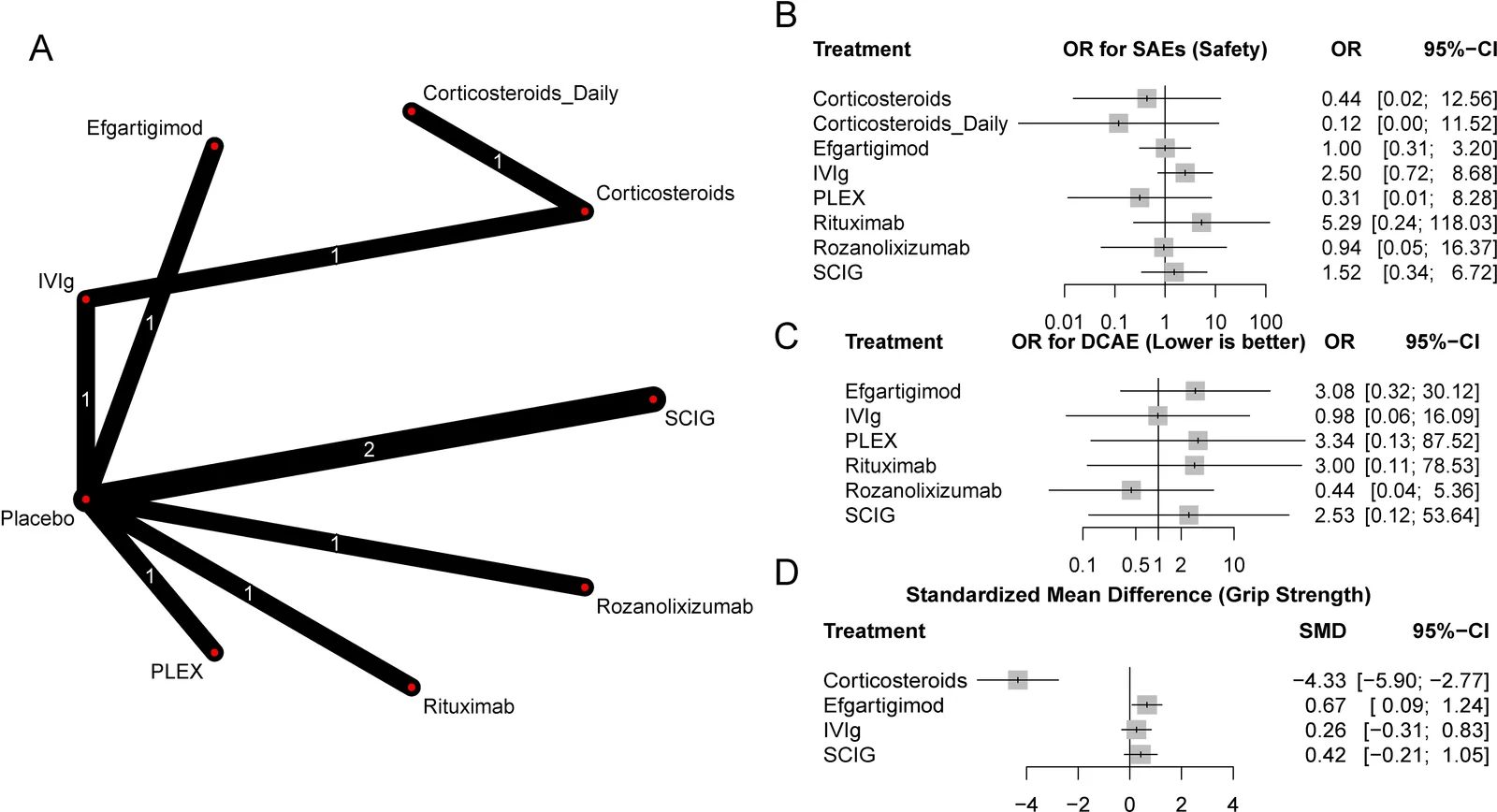
Safety, acceptability, and functional outcomes. **(A)** Network plot for Serious Adverse Events (SAEs). Illustrates the evidence structure for safety analysis. **(B)** Forest plot for SAEs. Relative risk of serious complications compared to Placebo. FcRn inhibitors (Efgartigimod, Rozanolixizumab) show a safety profile comparable to Placebo. **(C)** Forest plot for Treatment Acceptability (DCAE). Rates of discontinuation due to adverse events. Rozanolixizumab and IVIg exhibit the highest acceptability. **(D)** Forest plot for grip-strength change, expressed as standardized mean difference (SMD). Efgartigimod ranked highest for this distal motor outcome, but the sparse evidence base limits inference about overall functional recovery.

Treatment acceptability was measured by the rate of discontinuation due to adverse events (DCAE). The DCAE network showed no observed heterogeneity (I2 = 0.00%) and no significant inconsistency (Q-test P = 0.91). Rozanolixizumab (36) and IVIg (25, 34) achieved the highest acceptability scores (P-scores 0.7896 and 0.6089, respectively), while PLEX (21) and efgartigimod (37) showed moderate acceptability in the randomized phases (**Figure 3C**; Appendix Table 1).

### 3.5 Functional outcome: grip strength

Grip-strength change was synthesized from 5 studies and treated as a distal motor outcome. The grip-strength network showed modest heterogeneity (I2 = 24.10%) and no significant inconsistency (Q-test P = 0.45). Efgartigimod alfa (37) showed the largest estimated improvement versus placebo (SMD 0.67; 95% CI 0.09–1.24) and the highest P-score (0.8847). SCIG (SMD 0.42) and IVIg (SMD 0.26) showed smaller estimated improvements. Because the grip-strength evidence was sparse, involved different instruments and baseline weakness profiles, and did not consistently include multidimensional disability outcomes, this result should not be interpreted as proof of superior global functional recovery, nerve repair, axonal preservation, or disease modification (**Figure 3D**; **Supplementary Figure 5**; Appendix Table 1).

### 3.6 Integrated decision analysis

The 2D clustered ranking plots and radar charts (**Figure 4**) synthesized the multidimensional data. PLEX and corticosteroids were positioned in the high-efficacy/moderate-safety quadrant (**Figure 4A**). IVIg remained the most balanced conventional therapy across efficacy and acceptability (**Figure 4B**). Radar charts suggested that efgartigimod ranked favorably for grip-strength change, whereas rozanolixizumab ranked favorably for acceptability; these profiles should be interpreted as outcome-specific signals rather than evidence of comprehensive functional superiority (**Figures 4C, D**).

**Figure 4:**
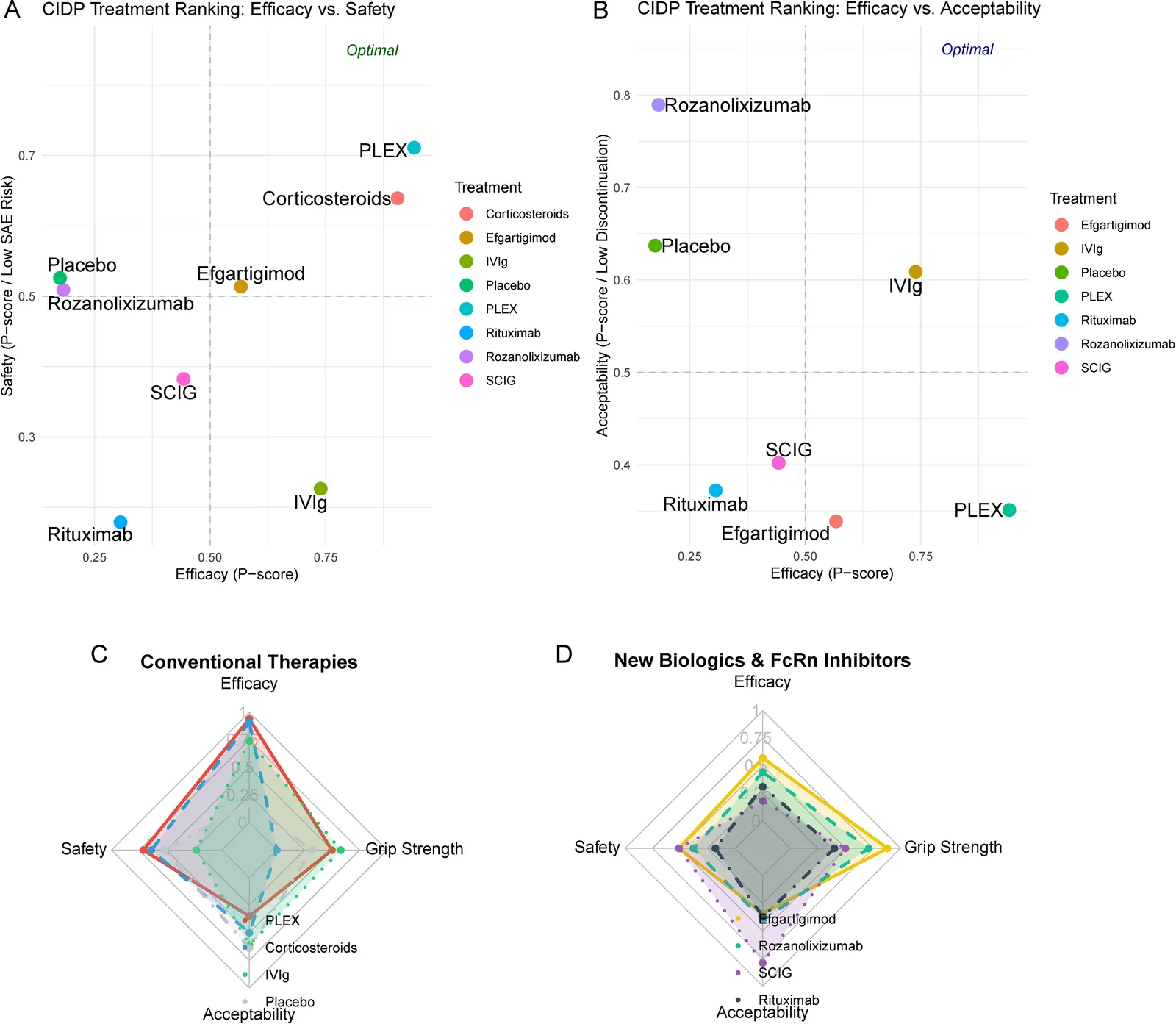
Integrated decision and multidimensional profiling. **(A)** 2D Clustered Ranking: Efficacy vs. Safety. Treatments in the upper-right quadrant (e.g., PLEX, Corticosteroids) provide the best balance between clinical response and low SAE risk. **(B)** 2D Clustered Ranking: Efficacy vs. Acceptability. Treatments like IVIg show high stability in both domains. **(C)** Radar Chart: Conventional Therapies. Multidimensional profiles of PLEX, IVIg, and Corticosteroids across four domains. **(D)** Radar Chart: New biologics and FcRn inhibitors. Outcome-specific profile of efgartigimod for grip-strength change and rozanolixizumab for acceptability.

### 3.7 Subgroup and sensitivity analysis

Subgroup analysis by clinical phase was interpreted cautiously because trial design and prior treatment exposure are partly confounded with intervention class. In the induction/active-treatment subgroup, PLEX (P-score 0.9183) and corticosteroids (0.8437) ranked highest. In the maintenance/withdrawal subgroup, high-dose IVIg and efgartigimod showed higher relative rankings. However, efgartigimod was classified according to the randomized withdrawal phase of ADHERE rather than as an induction trial, because only responders from the open-label phase entered the placebo-controlled randomized phase used in the network. Sensitivity analyses using broader classifications for mixed and active-deterioration studies did not alter the qualitative conclusion that conventional rapidly acting therapies dominate induction estimates, while maintenance estimates remain driven by IVIg/SCIG and FcRn-inhibitor withdrawal data (**Supplementary Figure 1**; Appendix Table 3).

Heterogeneity for the primary clinical-response outcome was extremely low (I2 = 0.37%), and inconsistency was not significant (Q-test P = 0.885); these values are now reported with the primary outcome and summarized with secondary-outcome I2 values in Appendix Table 4. The comparison-adjusted funnel plot showed general symmetry, suggesting no clear evidence of publication bias, although interpretation remains limited by the small number of studies per comparison (**Supplementary Figure 4**).

## 4 Discussion

This systematic review and network meta-analysis (NMA) compares conventional immunotherapies and newer biological agents, including neonatal Fc receptor (FcRn) inhibitors, for adult chronic inflammatory demyelinating polyradiculoneuropathy (CIDP). By incorporating pivotal trials through February 2026, including the ADHERE study (39), our analysis summarizes the current comparative evidence while recognizing that trial phase, enrichment design, and sparse treatment nodes limit the certainty of some indirect comparisons (40).

The primary efficacy analysis demonstrated that plasma exchange (PLEX) and corticosteroids remain the most potent interventions for achieving clinical response, particularly in treatment-naïve patients (41). PLEX exhibited the highest probability of being the most effective induction therapy (P-score 0.9415), likely due to its immediate and non-selective removal of circulating autoantibodies, complement components, and pro-inflammatory cytokines (42). Similarly, the high efficacy of corticosteroids (P-score 0.9056) underscores their robust ability to stabilize the blood-nerve barrier and suppress T-cell-mediated inflammation (43). However, the discordance between their high efficacy and lower acceptability scores (0.3511 and 0.5020, respectively) highlights a clinical reality: while these “legacy” therapies are effective in acute settings, their long-term utility is severely hampered by technical demands and systemic toxicity (44).

Efgartigimod alfa ranked highest for grip-strength change, despite a moderate ranking for global clinical response (P-score 0.5668 for response; P-score 0.8847 and SMD 0.67 for grip strength) (45). This finding is clinically relevant because grip strength is a responsive distal motor measure in CIDP, but it should be interpreted alongside disability scales, gait outcomes, relapse prevention, and patient-reported outcomes (46). The present analysis does not provide direct mechanistic or longitudinal neurophysiological evidence for superior nerve repair, prevention of axonal loss, or disease modification. A more conservative interpretation is that FcRn inhibition may produce a favorable distal-strength signal in the available randomized withdrawal data, warranting confirmation in trials with comprehensive functional and electrophysiological endpoints (47, 48).

Furthermore, our safety and acceptability analysis provides supportive information for long-term treatment selection. The SAE estimates for efgartigimod and rozanolixizumab were close to placebo in the randomized data (P-scores approximately 0.51), but confidence intervals were wide for sparse nodes (49). In terms of acceptability, rozanolixizumab ranked highest (P-score 0.7896), suggesting a favorable discontinuation profile in the available phase 2a data (50). In contrast, IVIg showed lower SAE ranking in this network, which should be interpreted in the context of trial populations, dose schedules, and known infusion-related and thromboembolic risks (51).

The subgroup analysis by clinical phase suggests, rather than confirms, distinct roles for therapies at different stages of treatment. In induction or active-treatment settings, PLEX and corticosteroids ranked highly for short-term response (52). In maintenance and withdrawal settings, estimates were driven by IVIg/SCIG continuation or withdrawal designs and by FcRn-inhibitor randomized withdrawal data (53). The ADHERE trial supports relapse-prevention efficacy for efgartigimod among patients who responded during the open-label stage (54), but these data should not be extrapolated directly to treatment-naive induction without dedicated randomized evidence (55).

The role of rituximab remains nuanced in our analysis. While its ranking in typical CIDP was lower (P-score 0.3063 for efficacy), recent evidence suggests that B-cell depletion is highly effective in patients with autoimmune nodopathies associated with anti-NF155 or anti-CNTN1 IgG4 antibodies (56). Since typical CIDP trials often include a mix of phenotypes, the efficacy of rituximab may be underestimated in general networks (57). Future clinical trials should focus on biomarker-stratified cohorts to better delineate the response to B-cell-targeted therapies versus FcRn antagonism (58).

This study has several limitations. First, outcome reporting varied across three decades of CIDP trials, and the use of standardized mean differences for grip strength may obscure clinically meaningful unit-level differences (59). Second, some nodes, especially rozanolixizumab and rituximab, were based on few trials and wide confidence intervals, leading to low or very low certainty for several comparisons (60). Third, crossover trials, small pilot studies, and randomized-withdrawal designs may introduce carry-over, imprecision, and responder-enrichment effects that limit transitivity. Fourth, grip strength was available in only five studies and cannot represent multidimensional functional prognosis on its own. Fifth, this NMA used aggregate published data and therefore could not evaluate patient-level effect modification by CIDP subtype, nodal/paranodal antibody status, prior treatment response, comorbidities, or biomarkers. Long-term real-world data and longitudinal electrophysiological outcomes for the newest biologics remain limited (61).

In conclusion, this network meta-analysis provides an aggregate-data comparison of CIDP treatments across efficacy, safety, acceptability, and grip-strength outcomes. PLEX and corticosteroids showed the strongest relative estimates for short-term response, IVIg remains a well-supported conventional therapy across several settings, and efgartigimod showed a favorable grip-strength signal with a safety estimate comparable to placebo in randomized withdrawal data. Rozanolixizumab showed favorable acceptability in a small phase 2a dataset. These findings support outcome-aware treatment selection, but individualized or precision-medicine recommendations require patient-level studies incorporating disability, relapse, gait, patient-reported, electrophysiological, and biomarker outcomes.

## Data Availability

The original contributions presented in the study are included in the article/**Supplementary Material**. Further inquiries can be directed to the corresponding author.

## Supplementary material

Supplementary Figure 5 Grip-strength evidence contribution by direct comparison. The figure shows the number of participants contributing extractable grip-strength change data in each direct comparison. It highlights the sparse nature of the grip-strength network and supports cautious interpretation of P-score rankings.

## Appendix

**Appendix Table 1:** Ranking of treatments based on P-scores across multiple outcomes.

Treatment	Efficacy (response)	Safety (SAEs)	Acceptability (DCAE)	Grip strength
PLEX	0.9415	0.711	0.3511	0.5012
Corticosteroids	0.9056	0.6393	0.502	0
IVIg	0.7392	0.2265	0.6089	0.5811
Efgartigimod	0.5668	0.5137	0.3388	0.8847
SCIG	0.4427	0.3825	0.4021	0.711
Rituximab	0.3063	0.1789	0.3723	0.4005
Rozanolixizumab	0.1825	0.5091	0.7896	0.5
Placebo	0.1752	0.5259	0.6372	0.3232

P-scores range from 0 to 1, where a higher value indicates a better profile for that specific outcome (higher efficacy, higher safety/lower SAEs, higher acceptability/lower drop-outs, and greater grip strength improvement).

**Appendix Table 2:** League table of primary efficacy.

PLEX	1.52 (0.42–5.51)	2.15 (0.85–5.43)	4.86 (1.42–16.63)	5.92 (1.65–21.21)	6.88 (1.52–31.12)	10.45 (2.05–53.25)	12.45 (2.34–66.10)
0.66 (0.18–2.38)	Corticosteroids	1.42 (0.60–3.35)	3.20 (0.95–10.75)	3.90 (1.08–14.12)	4.54 (1.02–20.24)	6.89 (1.35–35.15)	8.20 (1.95–34.50)
0.46 (0.18–1.18)	0.70 (0.30–1.67)	IVIg	2.26 (1.21–4.22)	2.75 (1.30–5.82)	3.20 (1.05–9.75)	4.86 (1.45–16.32)	5.78 (2.12–15.75)
0.21 (0.06–0.70)	0.31 (0.09–1.05)	0.44 (0.24–0.83)	Efgartigimod	1.22 (0.58–2.56)	1.42 (0.42–4.82)	2.15 (0.60–7.68)	2.56 (1.45–4.52)
0.17 (0.05–0.61)	0.26 (0.07–0.93)	0.36 (0.17–0.77)	0.82 (0.39–1.72)	SCIG	1.16 (0.35–3.85)	1.76 (0.50–6.15)	2.10 (1.10–4.02)
0.15 (0.03–0.66)	0.22 (0.05–0.98)	0.31 (0.10–0.95)	0.70 (0.21–2.38)	0.86 (0.26–2.86)	Rituximab	1.52 (0.35–6.62)	1.81 (0.55–5.95)
0.10 (0.02–0.49)	0.15 (0.03–0.74)	0.21 (0.06–0.69)	0.47 (0.13–1.67)	0.57 (0.16–2.00)	0.66 (0.15–2.86)	Rozanolixizumab	1.19 (0.38–3.75)
0.08 (0.02–0.43)	0.12 (0.03–0.51)	0.17 (0.06–0.47)	0.39 (0.22–0.69)	0.48 (0.25–0.91)	0.55 (0.17–1.82)		

Results are expressed as Odds Ratios (OR) with 95% Confidence Intervals. An OR > 1 indicates a higher probability of clinical response compared to the reference.

**Appendix Table 3:** Subgroup analysis: P-scores by clinical phase.

Treatment	Induction phase (naive)	Maintenance phase (withdrawal)
PLEX	0.9183	NA
Corticosteroids	0.8437	NA
IVIg	0.5703	0.7055
Efgartigimod	NA	0.5871
SCIG	NA	0.4415
Rituximab	0.1958	NA
Placebo	0.0889	0.1247

Comparison of treatment rankings between induction/active-treatment trials and maintenance/withdrawal trials. NA indicates that the treatment was not evaluated in the randomized data for that clinical subgroup. Efgartigimod was classified according to the randomized withdrawal phase of ADHERE.

**Appendix Table 4:** Heterogeneity and inconsistency assessment.

Outcome	Number of Studies	Heterogeneity(I²)	Inconsistency (Q-test P-value)
Clinical Response	11	0.37%	0.885
Safety (SAEs)	9	12.40%	0.642
Acceptability (DCAE)	7	0.00%	0.91
Grip Strength	5	24.10%	0.45

I^2^ values < 50% and P-values > 0.05 indicate low heterogeneity and high consistency between direct and indirect evidence, validating the NMA results.
