# Supplementary material for: Comparative efficacy, safety, and functional recovery of neonatal fc receptor inhibitors and conventional immunotherapies for chronic inflammatory demyelinating polyradiculoneuropathy: a systematic review and network meta-analysis
Source: Front Immunol. 2026 Sep 14;17:1856507. doi: 10.3389/fimmu.2026.1856507 (PMC13617617; doi:10.3389/fimmu.2026.1856507)
Supplement: Supplementary file 2 [file DataSheet2.zip › supplementary tableS5-S7/Supplementary_Tables_S5-S7.docx]

**Supplementary Tables**

**Supplementary Table S5. Potential effect modifiers assessed for transitivity.**

| **Domain** | **Distribution across trials** | **Implication for NMA interpretation** |
| --- | --- | --- |
| Trial era | Included trials were published from 1994 to 2026. | Older trials used smaller samples and more crossover designs; indirect comparisons involving older evidence were interpreted cautiously. |
| Treatment phase | Trials included treatment-naive/active-treatment, mixed, maintenance, and randomized-withdrawal designs. | Responder enrichment in withdrawal trials may inflate maintenance applicability and reduce direct comparability with induction trials. |
| Prior treatment exposure | Several maintenance trials enrolled IVIg responders or immunoglobulin-dependent patients. | Indirect comparisons with treatment-naive induction trials may violate exchangeability if prior response modifies treatment effect. |
| Baseline disability | Baseline scales varied across INCAT, ONLS, ODSS, MRC, and study-specific measures. | Functional and disability outcomes were not pooled as a single construct; grip strength was interpreted as a distal motor measure. |
| Study size | Some nodes were informed by small phase 2 or pilot trials. | Small nodes contributed imprecision and lower certainty, especially rozanolixizumab and rituximab. |
| Risk of bias | Most modern trials were low risk; earlier crossover and small trials raised some concerns. | Risk-of-bias concerns were incorporated into certainty judgments and discussion. |

Abbreviations: NMA, network meta-analysis; ODSS, Overall Disability Sum Score; MRC, Medical Research Council.

**Supplementary Table S6. Certainty-of-evidence summary using CINeMA/GRADE domains.**

| **Outcome/comparison** | **Within-study bias** | **Indirectness** | **Imprecision** | **Heterogeneity/incoherence** | **Overall certainty** | **Rationale** |
| --- | --- | --- | --- | --- | --- | --- |
| Clinical response: PLEX vs placebo/sham | Some concerns | Some concerns | Serious | Not serious | Low | Large effect but derived partly from older/small crossover evidence with wide CI. |
| Clinical response: corticosteroids vs placebo/standard comparators | Not serious | Some concerns | Serious | Not serious | Moderate to low | High ranking, but treatment regimens and comparators varied. |
| Clinical response: IVIg vs placebo | Not serious | Not serious | Not serious | Not serious | Moderate | Multiple trials with consistent direction of effect. |
| Clinical response: efgartigimod vs placebo | Not serious | Some concerns | Serious | Not serious | Moderate to low | Randomized withdrawal design enriches for responders; CI remains moderately wide. |
| Clinical response: rozanolixizumab vs placebo | Some concerns | Some concerns | Very serious | Not serious | Low to very low | Single small phase 2a dataset with wide CI. |
| Clinical response: rituximab vs placebo | Not serious | Some concerns | Very serious | Not serious | Low to very low | Single small trial; biomarker-defined responders not separable in aggregate data. |
| SAEs and DCAE across FcRn inhibitors | Not serious/some concerns | Some concerns | Serious | Not serious | Low | Few events and wide intervals; estimates should be interpreted descriptively. |
| Grip-strength change: efgartigimod and other nodes | Some concerns | Serious | Serious | Not serious | Low | Only five studies contributed data; grip strength is not a comprehensive functional endpoint. |

Certainty categories were assigned using CINeMA/GRADE principles for aggregate NMA evidence: within-study bias, reporting bias, indirectness, imprecision, heterogeneity, and incoherence. These judgments are intended to guide interpretation, not to replace a full patient-level certainty assessment.

**Supplementary Table S7. Grip-strength data contribution and measurement limitations.**

| **Study** | **Direct comparison** | **Participants contributing data** | **Comment** |
| --- | --- | --- | --- |
| Hahn 1996 (PLEX) | PLEX vs placebo/sham | 30 | Crossover design; older trial era; grip strength reported as impairment measure. |
| Hahn 1996 (IVIg) | IVIg vs placebo | 30 | Crossover design with short follow-up. |
| Merkies 2009 (ICE) | IVIg vs placebo | 117 randomized; grip SD available by arm | Vigorimeter grip strength is responsive but not equivalent to global disability. |
| Nobile-Orazio 2012 | IVIg vs IV methylprednisolone | 45 | Head-to-head conventional therapy comparison; no placebo arm. |
| Allen 2024 (ADHERE) | Efgartigimod vs placebo | 221 | Randomized withdrawal after Stage A response; supports maintenance/relapse-prevention context. |

Grip-strength analyses were downgraded because of sparse data, differences in measurement context, baseline weakness, prior treatment exposure, and lack of uniform linkage to INCAT, ONLS, I-RODS, gait, or patient-reported outcomes.
